# Algorithms for optimizing cross-overs in DNA shuffling

**DOI:** 10.1186/1471-2105-13-S3-S3

**Published:** 2012-03-21

**Authors:** Lu He, Alan M Friedman, Chris Bailey-Kellogg

**Affiliations:** 1 Dept of Computer Science, Dartmouth College, 6211 Sudikoff Laboratory, Hanover, NH 03755, USA; 2 Dept of Biological Sciences, Markey Center for Structural Biology, Purdue Cancer Center, and Bindley Bioscience Center, Purdue University, West Lafayette, IN 47907, USA

## Abstract

**Background:**

DNA shuffling generates combinatorial libraries of chimeric genes by stochastically recombining parent genes. The resulting libraries are subjected to large-scale genetic selection or screening to identify those chimeras with favorable properties (e.g., enhanced stability or enzymatic activity). While DNA shuffling has been applied quite successfully, it is limited by its homology-dependent, stochastic nature. Consequently, it is used only with parents of sufficient overall sequence identity, and provides no control over the resulting chimeric library.

**Results:**

This paper presents efficient methods to extend the scope of DNA shuffling to handle significantly more diverse parents and to generate more predictable, optimized libraries. Our CODNS (cross-over optimization for DNA shuffling) approach employs polynomial-time dynamic programming algorithms to select codons for the parental amino acids, allowing for zero or a fixed number of conservative substitutions. We first present efficient algorithms to optimize the local sequence identity or the nearest-neighbor approximation of the change in free energy upon annealing, objectives that were previously optimized by computationally-expensive integer programming methods. We then present efficient algorithms for more powerful objectives that seek to localize and enhance the frequency of recombination by producing "runs" of common nucleotides either overall or according to the sequence diversity of the resulting chimeras. We demonstrate the effectiveness of CODNS in choosing codons and allocating substitutions to promote recombination between parents targeted in earlier studies: two GAR transformylases (41% amino acid sequence identity), two very distantly related DNA polymerases, Pol X and *β *(15%), and beta-lactamases of varying identity (26-47%).

**Conclusions:**

Our methods provide the protein engineer with a new approach to DNA shuffling that supports substantially more diverse parents, is more deterministic, and generates more predictable and more diverse chimeric libraries.

## Background

The harnessing of DNA recombination *in vitro *has transformed protein engineering by enabling engineers, like nature, to sample sequence space more broadly than is allowed by point mutagenesis at individual residues. Recombination produces chimeras comprised of sequential fragments from parent genes, thereby bringing together sets of sequences that were previously active in the parental background, and are thus likely to be less disruptive than random ones. Chimeragenesis typically produces combinatorial libraries, and those chimeras with beneficial properties can be identified by large-scale genetic screening and selection.

**Figure 1:**
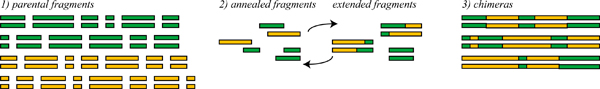
**Basic steps in gene shuffling protocol (following **[30]**).** (1) Parental genes are stochastically fragmented. (2) The fragments are denatured, and strands with sufficient complementarity are annealed and extended. Cross-overs are formed when the complementary strands are from different parents and can be extended to complete fragments. The process is repeated for multiple rounds, generating additional fragments and cross-overs. (3) Ultimately a chimeric library is generated, some of whose members represent full-length genes, as shown.

DNA shuffling [1,2], the progenitor of recombination-based protein engineering, works by randomly digesting the parent genes into fragments and reassembling the fragments into new chimeric genes (Figure 1). Recombination occurs when fragments from different parents are sufficiently complementary to anneal and prime synthesis from the 3' end. DNA shuffling has been called by its developer Pim Stemmer "the most dangerous thing you can do in biology" [3], due to its power in generating novel proteins. Indeed, it has been the basis both for commercial success (Affymax, Maxygen) and the development of effective protein variants [4-7].

DNA shuffling is both *homology-dependent *(recombination can occur only in runs of similar DNA sequence), and *stochastic *(the engineer does not control the recombination sites). Due to dependence on sequence similarity, DNA shuffling may fail to generate desirable chimeras (or any chimeras at all) for diverse parents, as they have only a few, small regions of DNA similarity, insufficient to generate many cross-overs. Homology-independent stochastic methods (e.g., ITCHY [8] and SHIPREC [9]) mitigate the need for such parental sequence similarity, but at the cost of generating many more non-viable chimeras.

In contrast with stochastic methods, site-directed methods enable the engineer to explicitly choose break-point locations so as to optimize expected library quality (e.g., by employing structural information [10], or by minimizing predicted disruption [11,12], library diversity [13], or both factors [14]). We have developed a site-directed method employing planned ligation of parental fragments by short overhangs [15]. We have coupled this approach to robotic implementation in order to generate specific chimeras in defined experimental vessels [16]. Such highly-directed methods of chimera generation are most useful when screening represents a significant effort. In those situations where screening or genetic selection is readily available, then stochastic approaches, with less overall cost, might prove preferable.

**Figure 2:**
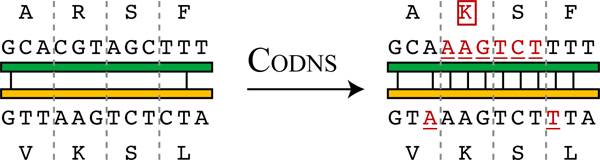
**Schematic overview of CODNS.** Here our method CODNS is applied to choose codons in a portion of two parental genes so as to produce a 9 nt "run" of common nucleotides, likely to be sufficient for a cross-over between complementary strands. To achieve the run, we use a combination of silent DNA substitutions (underlined) as well as a conservative amino acid substitution (boxed). The implications of these choices can be global; e.g., TTC for F and CTC for L would end the current run at 7 nt, but provide the first 2 nt of a new one. Our dynamic programming algorithm finds the globally optimal solution.

We present here methods for extending stochastic experiments by optimizing DNA shuffling (Figure 2), yielding an approach that is less dependent on parental DNA sequence similarity (parents can be more diverse) and more deterministic (cross-overs are more predictable), and which is amenable to library optimization. Our approach, which we call CODNS (cross-over optimization for DNA shuffling), employs efficient (polynomial-time) dynamic programming algorithms to select a globally optimal set of codons for the parental amino acids, allowing for a fixed number of substitutions. While Moore and Maranas have also studied the problem of codon optimization for shuffling [17], their eCodonOpt method employs computationally-expensive integer programming to select codons. We present dynamic programming recurrences for the two crossover-maximization objective functions of eCodonOpt: overall DNA sequence identity and overall free energy of annealing as approximated by a nearest-neighbor potential. We then develop recurrences for two more powerful objectives that seek to maximize crossovers by promoting DNA sequence identity within contiguous runs, optimizing either the overall number of runs or the diversity of the chimera library resulting from breakpoints in the contiguous runs.

We demonstrate the effectiveness of CODNS in several case studies. We first optimize the GAR transformylases previously optimized by eCodonOpt [17]. We then show that CODNS can optimize two DNA polymerases (Pol X and Pol *β*) that are sufficiently diverse (15% amino acid sequence identity) to previously require the development and application of the SCOPE method [10], instead of direct application of DNA shuffling. Finally, we study the impact of parental sequence identity by considering pairs of beta-lactamase parents of differing diversity levels.

## Methods

We take as input the amino acid sequences of the *parent *proteins to be shuffled, aligned to a length of *n *(amino acids and gaps) based on sequence and/or structure. For simplicity of exposition, we present our methods for the most common case of shuffling two parents, *a*_1 _and *a*_2_. Our methods readily extend to creating equivalent sites for recombination in multiple parents, and it remains interesting future work to allow for non-uniform shuffling (i.e., where different cross-overs are possible between different pairs of parents).

To optimize the shuffling experiment, we select a codon for each amino acid for each parent, yielding DNA sequences *d*_1 _and *d*_2 _of length 3*n *(maintaining gaps for those in the amino acid sequences). To expand the pool of codons being considered at a particular position, we may choose to make an amino acid substitution. Thus we take as additional input a specification of the *allowed substitutions *for each residue position for each parent, along with a number *m *of them to make. The allowed substitution specification may be derived from sequence and/or structural analysis of the parents, including general amino acid substitution matrices [18], position-specific amino acid statistics from related proteins [19], and $\Delta \Delta {\text{G}}_{\text{fold}}^{\circ }$ fold predictions for possible substitutions [20]. The results presented below determine allowed substitutions under the BLOSUM62 substitution matrix, considering only "conservative" substitutions which score no more than 4 worse than wild-type [15].

In describing the algorithms, we use *possible codon sets *representing the codons allowed at each position in the wild-type and under the allowed substitutions. For position *i*, set *C*_1_[*i*] contains the possible codons for *a*_1_[*i*], pairing each with an indication of whether or not it requires a substitution, e.g., {(TTT, 0), (TTC, 0), (TGG, 1)} for an F that could potentially be mutated to W. Set *C*_2_[*i*] is defined similarly for the second parent. We note that these may readily be used to restrict where to employ mutations (e.g., masking based on structural analysis, as discussed by Moore and Maranas [17]), by allowing only wild-type codons (or amino acids) in some positions.

We consider four types of objective function, targeting *common nucleotides *(at aligned positions), *nearest-neighbor approximation to change in free energy of annealing *(from dinucleotide pairs), *common nucleotide runs *(in contiguous strings), or *library diversity *(among resulting chimeras). We develop increasingly more complex dynamic programming algorithms to optimize these objectives.

### Common nucleotide optimization

In this most basic optimization for DNA shuffling, the goal is to maximize the number of identical nucleotides at common positions:

(1) $$o_{\text{nt}}=\overset{3n}{\underset{i=1}{\sum }}I\left\{d_{1}\left[i\right]=d_{2}\left[i\right]\right\}$$

where *I *is the indicator function (1 for true, 0 for false).

With no substitutions allowed, each residue position is independent of each other one. Thus we simply select for each position a pair of codons (one for each parent) with a maximal number of common nucleotides. When substitutions are allowed, we need to allocate them for optimal impact. While several approaches are possible, we develop here one based on dynamic programming, to serve as the basis for the more complex objective functions we pursue in subsequent subsections.

In our dynamic programming matrix, one dimension represents an aligned residue position (i.e., we have optimized the sequences up to that point), and the other represents a number of substitutions (i.e., we have made that many thus far). Let *N*[*i*, *s*] denote the number of common nucleotides within the first *i *residues, using exactly *s *substitutions. The value of *N*[*i*, *s*] extends the value of *N*[*i *- 1, *s *- (*t*_1 _+ *t*_2_)] with the additional number of common nucleotides obtained by selecting a pair of codons for position *i *while making *t*_1 _+ *t*_2 _additional substitutions (0 or 1 for each parent). Optimal substructure holds, since the optimal value of *N*[*i*, *s*] depends on the optimal value of *N*[*i *- 1, *s *- (*t*_1 _+ *t*_2_)]. The recurrence is

(2) N[0,0]=0

(3) N[0,s]=-∞s>0

(4) $$N\;[i,\;s]=\underset{\begin{array}{l} (c_{1},t_{1})\in C_{1}[i],\\ \underset{t_{{}_{1}}+t_{2}\leq s}{(c_{2},t_{2})\in C_{2}[i]:} \end{array}}{\max}\;(N\;[i-1,\;s-(t_{1}+t_{2})]+g(c_{1},\;c_{2}))\;i>0,\;s>0$$

where *g *gives the number (0-3) of common nucleotides for a pair of codons.

After filling in the dynamic programming table, we trace back from *N*[*n*, *m*] to generate an optimal pair of DNA sequences. The matrix is of size n * *m *and each cell takes constant time to compute.

### $\Delta {\text{G}}_{\text{nn}}^{\circ }$ optimization

While it is hard to directly model the process of DNA shuffling [21-23], it is driven by the change in free energy upon annealing of the different parental nucleotide strands (coding of one with non-coding of the other; see Figure 1). We want to minimize the free energy change, so that it is favorable to cross over. Since the free energy change is very hard to compute, a common approach is to approximate it by decomposing the free energy into the sum of contributions from pairs of dinucleotides, the *nearest-neighbor *approximation [24]:

(5) $$o_{\text{nn}}=\overset{3n-1}{\underset{i=1}{\sum }}\Delta {\text{G}}_{\text{nn}}^{\circ }\left(d_{1}\left[i\right]\cdot d_{1}\left[i+1\right],\;d_{2}\left[i\right]\cdot d_{2}\left[i+1\right]\right)$$

where $\Delta {\text{G}}_{\text{nn}}^{\circ }\left(d_{1}\left[i\right]\cdot d_{1}\left[i+1\right],\;d_{2}\left[i\right]\cdot d_{2}\left[i+1\right]\right)$ is the free energy change associated with annealing dinucleotide *d*_1_[*i*]·*d*_1_[*i *+ 1] with dinucleotide *d*_2_[*i*]·*d*_2_[*i *+ 1]. These values can be computed from enthalpic Δ*H *(kcal/mol) and entropic Δ*S *(cal/mol·K) nearest-neighbor parameters compiled at 37°C and [Na^+^] = 1.0 M [24], including both pairs of complementary strands. To actually estimate the change in free energy, there are additional constant terms such as the average initiation energy contribution; we omit them as they do not affect the optimization. While the underlying $\Delta {\text{G}}_{\text{nn}}^{\circ }$ parameters are defined on pairs of dinucleotides, we abuse notation a bit in our formulation below and use $\Delta {\text{G}}_{\text{nn}}^{\circ }$ for 4-mers to mean the sum over the constituent dinucleotides.

**Figure 3:**
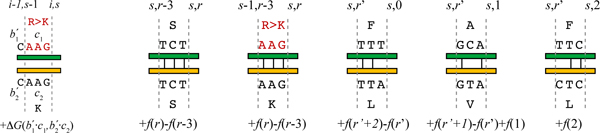
**Examples of dynamic programming recurrences.** The top part of each example shows relationships in the table and the bottom part the score differences. (left) $\Delta {\text{G}}_{\text{nn}}^{\circ }$ optimization, a nearest-neighbor approximation to the change in free energy of annealing, summing adjacent nucleotide pairs. We must keep track of the third nucleotides of the codons for position *i *- 1 in order to compute the $\Delta {\text{G}}_{\text{nn}}^{\circ }$ contribution. (right) Run optimization cases: common codon; common codon after substitution; continue and end a run; continue and end one run and start a new one; end one run and start a new one. The run score contribution is computed based on the pattern of nucleotide matches.

We now develop a dynamic programming formulation to optimize this objective more efficiently (in polynomial time) than the integer linear programming of eCodonOpt [17], while still ensuring global optimality. In order to compute the $\Delta {\text{G}}_{\text{nn}}^{\circ }$ contributions from a selected codon, we must also know the final nucleotide of the previous codon, as it forms a dinucleotide with the first nucleotide of the current codon. Thus we extend the common nucleotide dynamic programming table to keep track of this information. Figure 3(left) illustrates the dependency; the recurrence is

(6) $$A\;\left[0,0,\;b_{1},\;b_{2}\right]=0$$

(7) $$A\;\left[0,\;s,\;b_{1},\;b_{2}\right]=\infty \;s>0$$

(8) $$A\;[i,\;s,\;b_{1},\;b_{2}]=\underset{\begin{array}{l} (c_{1},t_{1})\in C_{1}[i],\\ \underset{\underset{t_{1}+t_{2}\leq s}{b_{1}=c_{1}[3],b_{2}=c_{2}[3],}}{(c_{2},t_{2})\in C_{2}[i]:} \end{array}}{\min}\;\;\underset{{b^{\prime }}_{1},\;{b^{\prime }}_{2}}{\min}\;(A\;[i-1,\;s-(t_{1}+t_{2}),\;{b^{\prime }}_{1},\;{b^{\prime }}_{2}]+\Delta {\text{G}}_{\text{nn}}^{\text{°}}({b^{\prime }}_{1}\cdot c_{1},\;{b^{\prime }}_{2}\cdot c_{2}))\;i>0,\;s>0$$

where *b*·*c *indicates the concatenation of base *b *onto codon *c*, and $\Delta {\text{G}}_{\text{nn}}^{\circ }$ estimates the change in free energy, as described in the text. Cell *A*[*i*, *s*, *b*_1_, *b*_2_] holds the best score for the first *i *positions, using exactly *s *substitutions, with third nucleotides *b*_1 _(first parent) and *b*_2 _(second parent) for position *i*. As with common nucleotide optimization, if a codon pair makes *t*_1 _+ *t*_2 _substitutions at position *i*, then *A*[*i*, *s*, *b*_1_, *b*_2_] extends the solution to a cell for position *i *- 1 with *s *- (*t*_1 _+ *t*_2_) substitutions, considering any of the third nucleotides $b_{1}^{\prime }$ and $b_{2}^{\prime }$ at position *i *- 1.

The table is of size *n *× *m *× 5^2 ^for 2 parents, since there are only (4 + 1)^2 ^combinations of single nucleotide pairs for two parents (four nucleotides and a gap each). Each cell can be computed in constant time. In practice, we construct a 2D table (over *i *and *s*), with each cell maintaining a list of scores for the (*b*_1_, *b*_2_) pairs that actually occur.

### Run optimization

Moore and Maranas argued that the nearest-neighbor approximation to change in annealing free energy is a better objective for shuffling optimization than the number of common nucleotides [17]. Intuitively, since the nearest-neighbor approximation considers adjacent nucleotides together rather than treating them independently, it is more likely to yield sufficient complementarity between fragments and thereby promote recombination. Here we go even further and explicitly optimize for contiguous complementary regions, since annealing is driven by sufficiently long (anecdotally 6 nt or more) such regions.

We define a *common nucleotide run *as a maximal-length substring appearing at aligned positions in the DNA sequences *d*_1 _and *d*_2_, and use as our objective function:

(9) $$o_{\text{run}}=\underset{R}{\sum }f\left(\left|R\right|\right)$$

where *f*, which must be non-decreasing, indicates the value for DNA shuffling of a run of length |*R*|, and the sum is taken over all runs. We have implemented and tested several different scoring functions; the results use the following two functions:

(10) $$f_{1}\left(r\right)=\left\{\begin{array}{c} 0\;\;r<\theta \\ r\;\;r\geq \theta \end{array}\right)$$

(11) $$f_{2}\left(r\right)=\left\{\begin{array}{cc} 0 & r<6\\ 9/4*\left(r-5\right) & 5\leq r<9\\ r & r\geq 9 \end{array}\right)$$

In *f*_1_, we count the total number of nucleotides in a run, but only if the run exceeds a given length (we empirically evaluated several thresholds). This assumes that cross-overs are impossible for runs with fewer than *θ *common nucleotides, and become increasingly likely with additional nucleotides beyond *θ*. In *f*_2_, we consider cross-overs impossible for fewer than 6 nucleotides and very likely for 9 nucleotides or more (scoring the total number of nucleotides as in *f*_1_), and we ramp up from the impossible score of 0 at 5 nt to the likely score of 9 at 9 nt, thereby counting the partial benefit that may be provided by runs between 6 and 9 nucleotides.

We must extend our dynamic programming table with an additional dimension to keep track of the current run length. Thus we have a table in which cell *R*[*i*, *s*, *r*] holds the best score for the first *i *positions, using exactly *s *substitutions, such that the final nucleotide in the codons chosen for position *i *is the *r*th in a run (0 if mismatch). Again, if we make *t *substitutions at position *i*, then *R*[*i*, *s*, *r*] extends the solution to a cell for position *i *- 1 with *s *- (*t*_1 _+ *t*_2_) substitutions. Now we must also account for the preceding run length; there are several cases (Figure 3, right): the codons chosen for the current amino acid position may continue a run from the previous position, may end that run, and may start a new run. In any case, the current *r *and possible codon pair determines the preceding *r*' at which to look, and optimal substructure still holds. The recurrence is thus

(12) R[0,0,0]=0

(13) R[0,s,r]=-∞s>0orr>0

(14) $$R[i,\;s,\;r]=\underset{\begin{array}{l} (c_{1},t_{1})\in C_{1}[i],\\ \underset{t_{1}+t_{2}\leq s}{(c_{2},t_{2})\in C_{2}[i]:} \end{array}}{\max}\{\begin{array}{ll} R[i-1,\;s-(t_{1}+t_{2}),\;r-3]+f(r)-f(r-3) & c_{1}=c_{2},\;r\geq 3\\ \begin{array}{l} \underset{r^{\prime }}{\max}(R[i-1,\;s-(t_{1}+t_{2}),\;r^{\prime }]\\ \;\;+f(r^{\prime }+a(c_{1},\;c_{2}))-f(r^{\prime })+f(r))\;r=z(c_{1},\;c_{2})<3 \end{array} & \\ -\infty & \text{otherwise} \end{array}i>0$$

where *a*(*c*_1_, *c*_2_) and *z*(*c*_1_, *c*_2_) give the lengths of the longest common prefix and suffix, respectively, of a pair of codons. The first case handles a common codon, while the second case handles an unequal codon pair, which may end and/or begin a run. The score depends on that from the related cell, with an increment in *f*(·) accounting for any extension in run length and initiation of a new run. (See again Figure 3, right.) When there is a tie, we prefer the codon pair with the most common nucleotides, even if that has no impact on run score. This choice increases overall sequence identity, to promote better annealing of strands from different parents.

The matrix is of size n * m * (3*n *+ 1), since the run length potentially ranges from 0 to the entire DNA sequence length (3*n*). However, in practical cases, most run lengths are not attainable. Furthermore, for *r*_1 _*< r*_2_, if *R*[*i*, *s*, *r*_1_] + *f*(*r*_2_) *- f*(*r*_1_) *< R*[*i*, *s*, *r*_2_], then the *r*_2 _cell "dominates" the *r*_1 _one--the *r*_1 _one cannot be part of the optimal solution. Thus we modify the usual dynamic programming algorithm slightly, to avoid filling in cells with unattainable or dominated run lengths. We perform the standard nested loop over *i *(residue position) and *s *(number of substitutions). Then for each *i *and *s *we determine which run lengths are attainable and undominated and fill in only those entries. Rather than keeping a 3D table, we keep a 2D table in which each cell has a list of run lengths and their scores. Note from the structure of Eq. 14 that we can determine the run lengths for *i*, *s *from the possible codons at *i *and the run lengths that were attained and undominated for *i *- 1 and *s*, *s *- 1, and *s *- 2 (depending on the numbers of substitutions required for the codons).

### Diversity optimization

We have previously developed methods for optimizing the diversity of libraries of chimeras produced by site-directed recombination [13,14]. We showed that the total number of mutations in a library is a constant determined only by the parents, but that by assessing the squared-differences in the numbers, we can optimize for a relatively uniform sampling of sequence space. In the case of two parents, we define the *diversity variance *over a library as:

(15) $$o_{\text{div}}=\frac{1}{2^{\lambda }(2^{\lambda }-1)}*\overset{2^{\lambda }-1}{\underset{i=1}{\sum }}\overset{2^{\lambda }}{\underset{j=i+1}{\sum }}{\left(m(H_{i},\;H_{j})-\bar{m}\right)}^{2}$$

where *λ *is the number of fragments, *m*(*H_i_*, *H_j_*) is the *mutation level *(number of amino acid differences) between a pair of chimeras *H_i _*and *H_j_*, and $\bar{m}$ is the average of *m *over the library. (We drop a constant factor of 2, which doesn't affect the optimization.) To mitigate the effect of neutral mutations, rather than using literal equality we measure *m *using one of the standard sets of amino acid classes. The goal is to minimize the variance, seeking to sample sequence space as uniformly as possible.

The objective function is defined in terms of the chimeras in the library. In the context of DNA shuffling, we assume that a sufficiently large run of common nucleotides (with respect to a threshold *θ *as in Eq. 10) results in a breakpoint, and thus that the (full-length) chimeras are well-defined as all combinations of fragments between the breakpoints. Breakpoints resulting from smaller runs only add to the diversity of the resulting library.

For an efficient algorithm, we must be able to compute the objective function during the optimization, without enumerating the exponential number of chimeras. In our previous site-directed work [13], we developed a recursive formulation relating the diversity variance for a library to that of a sub-library with one fewer breakpoint. That formulation took as given the total number of breakpoints, which isn't available in the DNA shuffling context. However, similar algebraic manipulations (omitted due to lack of space) yield a related formula without requiring pre-knowledge of the number of breakpoints.

**Claim 1 ***The diversity variance d*(*l*, *k*) *of a library from parent sequences P_a _and P_b _with kth breakpoint is at residue l can be computed from the diversity variance d*(*l*', *k - *1) *for a library with *(*k *- 1) *st breakpoint at residue l*' *< l by the following formula:*

(16) $$d\left(l,\;k\right)=\frac{2^{k}-2}{2^{k}-1}*d\left(l^{\prime },\;k-1\right)+E\left(l,\;l^{\prime },\;k\right)$$

where

$$\begin{array}{ll} E(l,\;l^{\prime },\;k)= & \\ & \frac{2^{2(k-1)}}{2^{k}(2^{k}-1)}\begin{array}{c} \left(m(P_{a}[1,\;l^{\prime }],\;P_{b}[1,\;l^{\prime }])*m(P_{a}[l^{\prime }+1,\;l],\;P_{b}[l^{\prime }+1,\;l]\right)\\ \;+m{\left(P_{a}[l^{\prime }+1,\;l],\;P_{b}[l^{\prime }+1,\;l]\right)}^{2}) \end{array}\\ & +\frac{2^{k}-2}{2^{k}-1}*\frac{2*m{\left(P_{a}[1,l^{\prime }],P_{b}[1,l^{\prime }]\right)}^{2}*2^{2k-6}}{{\left(2^{k-1}-1\right)}^{2}}\\ & -\frac{2*m{\left(P_{a}[1,l],P_{b}[1,l]\right)}^{2}*2^{2k-4}}{{\left(2^{k}-1\right)}^{2}} \end{array}$$

*and we use notation P*[*i*, *j*] *to indicate the substring from position i to j, inclusive*.

Based on Eq. 16, we further extend our run-length optimization dynamic programming recurrence to optimize for diversity:

(17) D[0,0,0,0,0]=0

(18) D[0,s,r,l,k]=0s>0orr>0orl>0ork>0

(19) $$\begin{array}{r} D[i,s,r,l,k]=\underset{\begin{array}{c} (c_{1},t_{1})\in C_{1}[i],\\ (c_{2},t_{2})\in C_{2}[i]:\\ t_{1}+t_{2}\leq s \end{array}}{\min}\{\begin{array}{l} \begin{array}{l} D[i-1,\;s-(t_{1}+t_{2}),\;r-3,\;l,\;k]\\ \;\;-E(i-1,\;l,\;k+1)+E(i,\;l,\;k+1) \end{array}\\ \begin{array}{cc} \underset{\begin{array}{c} r^{\prime }+a(c_{1},c_{2})\geq \theta ,\\ l^{\prime }<i-1 \end{array}}{\min} & \begin{array}{l} \frac{2^{\left(k+1\right)}-2}{2^{\left(k+1\right)}-1}D\;[i-1,\;s-(t_{1}+t_{2}),\;r^{\prime },\;l^{\prime },\;k-1]\\ \;+E(i,\;i-1,\;k+1) \end{array} \end{array}\\ \begin{array}{cc} \underset{r^{\prime }+a(c_{1},c_{2})<\theta }{\min} & \begin{array}{l} D\;[i-1,\;s-(t_{1}+t_{2}),\;r^{\prime },\;l,\;k]\\ \;\;-E(i-1,\;l,\;k+1)+E(i,\;l,\;k+1) \end{array} \end{array}\\ \infty \end{array}\;\;\begin{array}{r} \begin{array}{l} c_{1}=c_{2},\;r\geq 3 \end{array}\\ \begin{array}{l} r=z(c_{1},\;c_{2})<3,\;l=i-1 \end{array}\\ \begin{array}{l} r=z(c_{1},\;c_{2})<3,\;l<i-1 \end{array}\\ \text{otherwise} \end{array}\\ i>0 \end{array}$$

We add two more dimensions, to keep track of *k*, how many runs of length *θ *we have seen (i.e., confidently yielding breakpoints), and *l*, where the last one was, as in the claim. Intuitively these two additional dimensions are necessary since the number of breakpoints affects the size of the library and thus the diversity variance, and since the additional diversity induced by a run depends on the nucleotides between the previous breakpoint and the new one. Note that in Eq. 16, *k *is the number of breakpoints, with the last breakpoint always at the end of the current position *l*; however, in Eq. 19, *k *is the number of previous runs, or *k *+ 1 when substituted into Eq. 16. As with run optimization, our implementation avoids filling in the table for run lengths that are unattainable (though the notion of dominated entries does not carry over).

### Codon usage

In order to promote better protein expression, we follow the GeneDesigner protocol [25] in employing organism-specific codon usage tables. A codon usage table for an organism [26] encodes the frequency with which each codon has been observed in a sequence database; different organisms display different "preferences" [27]. In a preprocessing step, we disallow rare codons that make up less than 10% of the occurrences for their amino acid. Then when computing one of the recurrences, we use the codon usage table to resolve cases where multiple possible codons give the same score (i.e., they have the same implications for continuing, ending, and beginning runs). In such cases, we selecting among the possible codons with probability according to their usage frequency.

## Results and discussion

We use three case studies to demonstrate the effectiveness of CODNS in optimizing DNA shuffling experiments. The first two case studies are a pair of glycinamide ribonucleotide (GAR) transformylases (previously optimized by eCodonOpt [17]) and a pair of distantly related DNA polymerases (previously recombined by SCOPE [10]). We optimize shuffling plans using from 0 to 10 mutations under each of the objective functions, abbreviated in the figures as *cn *(common nucleotides), Δ*G *(nearest-neighbor approximation to change in free energy of annealing), *f*_1 _(runs under *f*_1 _scoring), *f*_2 _(runs under *f*_2 _scoring), and *dv *(library diversity). We examine particular plans optimized under different objectives, in order to see how they differ in allocating mutations and producing homologous runs suitable for cross-overs. We then study the overall trends in optimizing the objectives and in producing runs. We also consider the diversity of the chimeras that would result by recombination under different run-optimal plans. Comparisons with what would result eCodonOpt [17] can be made by noting that it optimizes *cn *and Δ*G *(though we use an efficient dynamic programming algorithm to do so). In a third case study, we evaluate the effects of wild-type sequence identity on the optimization, using different pairs of beta-lactamases.

### GAR transformylases

The parents for our first case study are a GAR transformylase from *E. coli *and one from humans. Previous work showed that DNA shuffling crossovers are extremely rare without codon optimization [17]. We obtained the (gapless) alignment from the supplementary material of [17], and transcribed it to 201 amino acids with 82 (40.8%) in common. The wild-type DNA sequences had 47% nucleotides in common [17], with only two runs of length 7 and no runs longer than 7 nt.

**Figure 4:**
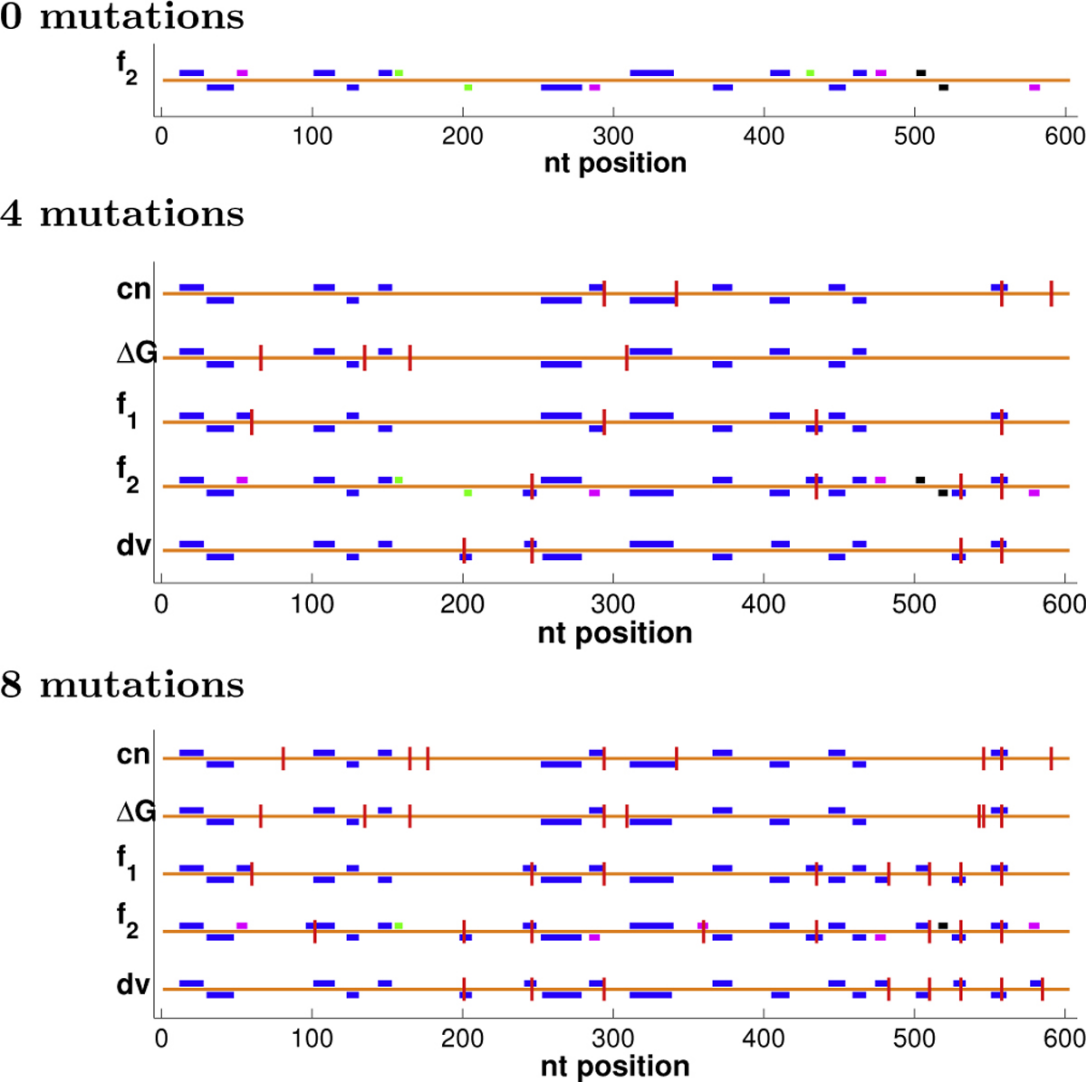
**GAR transformylase detailed plans.** Shown are the locations of selected mutations (vertical lines) and resulting runs of length ≥ 9 (boxes; staggered at some locations to avoid overlap), optimized under different objectives. For *f*_2_, runs are colored by length, with 6:green, 7:black, 8:magenta, ≥ 9:blue. For 0 mutations, all methods yielded the same run pattern, so only the *f*_2 _version is shown.

Figure 4 illustrates some optimal plans, showing runs of length ≥ 9, a relatively confident threshold for crossovers (analogous observations can be made with other thresholds, not shown to save space). In some cases, many plans may be tied for optimal under the objective. We extended our dynamic programming back-trace to generate the tied solutions [28]; for common nucleotides, we enumerated all, but for $\Delta {\text{G}}_{\text{nn}}^{\circ }$, we stopped after 1000 were generated. The figure then shows the tied-for-optimal plans with the most nucleotides in runs (at a threshold of 9). All objectives yield the same run patterns with 0 mutations. However, with more mutations, run and diversity optimization methods focus their efforts on regions that are sufficiently similar to allow formation of runs with well-placed mutations and well-chosen codons, while choices made by common nucleotide and $\Delta {\text{G}}_{\text{nn}}^{\circ }$ optimization are not as productive. Diversity optimization produces the same number of runs as *f*_1 _optimization, but places the runs more evenly throughout the entire sequence so that crossing over at those sites would yield chimeras comprised of more uniformly-sized fragments better sampling the sequence space spanned by the parents. ("Size" in diversity optimization refers to residues at which the parents differ, not just the total number of amino acids [13].)

**Figure 5:**
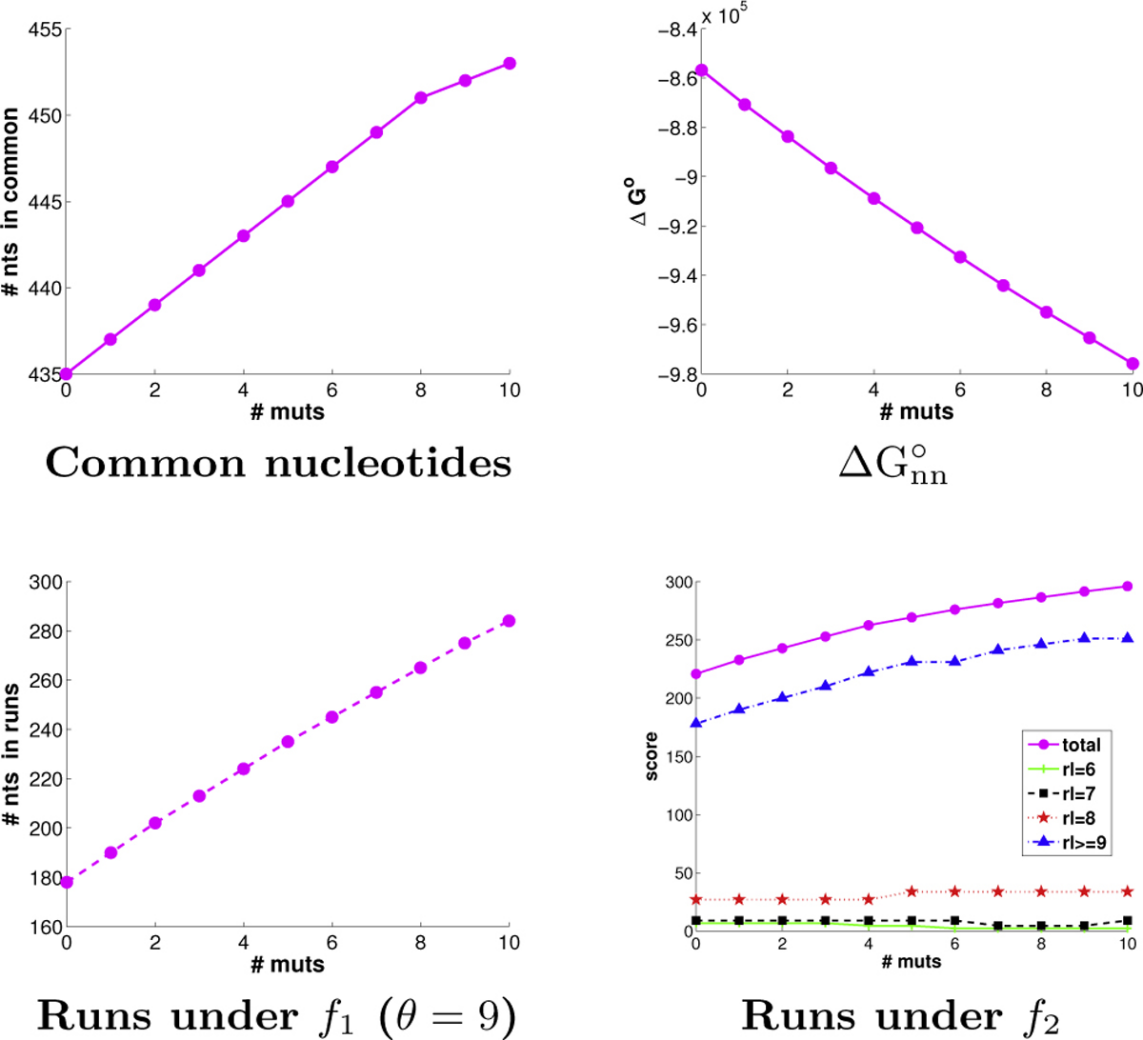
**GAR transformylase scoring trends.** The trends are given for each objective, from 0 to 10 mutations.

We next analyzed the overall ability of CODNS to select codons and allocate mutations to meet the different optimization goals. Figure 5 illustrates the objective score trends with increasing numbers of mutations. All of the plots are quite linear (recall that $\Delta {\text{G}}_{\text{nn}}^{\circ }$ is to be minimized), demonstrating that there is sufficient freedom within these two parents to enable effective optimization for the objectives, and that the algorithms are successfully exploiting the available freedom. Since the *f*_2 _metric gives "partial credit" for run lengths of 6, 7, and 8, we break out those contributions to its score. We see most of the optimization still focuses on full 9 nt and larger runs, which is natural given the reduced score contribution for shorter runs (since they are believed to be less productive in promoting recombination). The trends for diversity optimization are not shown here since scores are not directly comparable for libraries of different sizes (resulting from different numbers of runs yielded by different patterns of codons and mutations).

**Figure 6:**
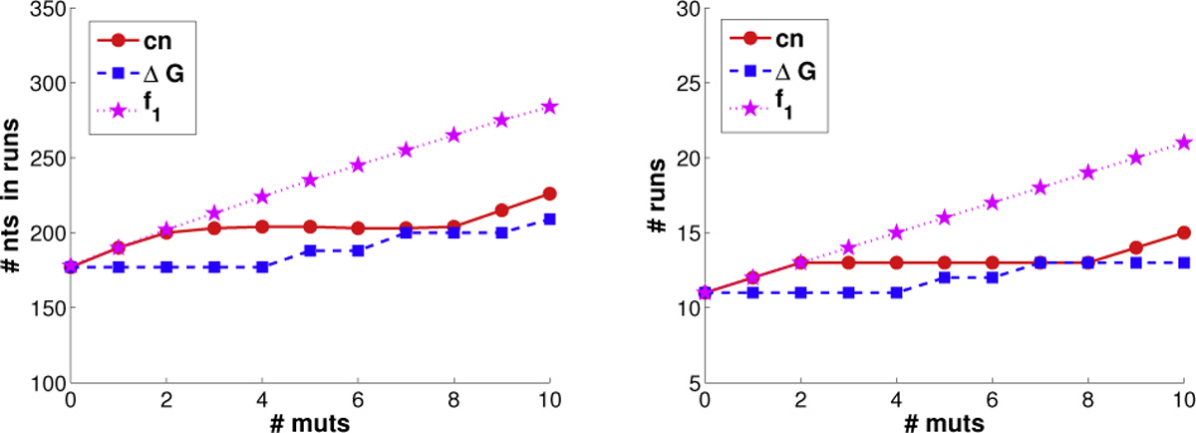
**GAR transformylase run trends.** Shown are the run trends (left: # nts in runs; right: # runs) in GAR transformylase plans employing from 0 to 10 mutations, optimized for common nucleotides (red), $\Delta {\text{G}}_{\text{nn}}^{\circ }$ (blue), and runs under *f*_1 _score (magenta).

We introduced novel run-based objective functions in order to more directly target the sufficient stretches of parental homology required for annealing, only indirectly optimized by the objectives of common nucleotides and $\Delta {\text{G}}_{\text{nn}}^{\circ }$ employed by eCodonOpt. To assess the impact of this more direct objective functions, we determined the number of runs produced by plans under the different objectives. We varied the threshold to consider a homologous region as a "run" from 7 (lower confidence) to 12 (higher confidence). As discussed above, among the plans tied for optimal for a particular objective, we sought the one with the best run score. We evaluated both the number of runs and the number of nucleotides in those runs. For the sake of space, Figure 6 presents only the results for a threshold of 9; the trends at the other thresholds are very similar. We see that, as mutations are introduced, optimizing directly for runs is indeed much more effective at producing runs than either of the "proxies" of common nucleotides or $\Delta {\text{G}}_{\text{nn}}^{\circ }$. We do not show trends for *f*_2_, as it also optimizes for "partial credit" runs (of lengths 6, 7, and 8).

**Figure 7:**
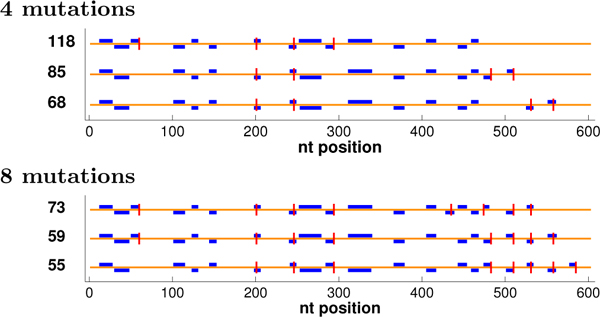
**GAR transformylase plans of different levels of diversity.** The plans are shown as in Figure 4, with different levels of diversity variance (noted on the left; smaller is better; the bottom one has the optimal score) but the same optimal number of runs (at *θ *= 9).

The final question regards diversity--how much control we can exert over the level of diversity we introduce (how different the resulting chimeras in a plan are from the parents and from each other). Here we deem a 9 nt run as sufficient for a breakpoint, and evaluate the ability of CODNS to minimize our library diversity variance objective (Eq. 15) while maximizing the number of runs. Figure 7 illustrates some plans with the same (optimal) number of runs but different library diversity scores. As discussed above, mutations in diversity-optimal plans are optimally allocated so as to create runs more evenly distributed throughout the entire sequence (counting positions with different amino acids in the parents). However, plans with larger diversity variance scores place runs closer together and leave the C-terminal portion without any runs, thereby generating no diversity there (the final 100+ residues will be from one parent or the other, rather than a hybrid).

### DNA polymerases

Our second case study involves two distantly-related members from the X-family of DNA polymerases: African swine fever virus DNA polymerase X (Pol X) and *Rattus norvegicus *DNA polymerase beta (Pol *β*). While these two proteins share a similar fold, they have very low sequence identity. The site-directed SCOPE method [10] was developed due to the difficulty in producing viable Pol X -- Pol *β *chimeras by other methods. We obtained the published structure-based sequence alignment of the two parents, in which the full Pol X and the palm and finger domains of Pol *β *were aligned to a length of 214 residues and gaps, with only 32 residues (15%) in common. The wild-type DNA sequences had only 158/642 (24%) nucleotides in common, with no common nucleotide runs of length greater than 5. Thus standard DNA shuffling techniques are unlikely to produce any cross-overs.

**Figure 8:**
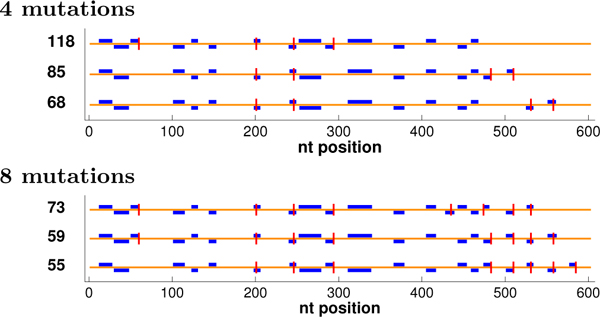
**DNA polymerase detailed plans.** The plans for the DNA polymerases are shown as in Figure 4.

We optimized these parents under each of our objective functions, using from 0 to 10 mutations. Figure 8 illustrates an optimal plan for each objective with 0, 4, or 8 mutations, showing runs of length ≥ 9. These particular parents are so diverse that only a few such runs can be produced by codon selection alone (no mutations). We do see, however, that the run-optimization methods form one more run (positions 148-157) than do the common nucleotide and $\Delta {\text{G}}_{\text{nn}}^{\circ }$ methods, and *f*_2 _forms some potentially productive shorter runs. The difference increases with more mutations, as run optimization directly allocates them so as to produce more runs, while, due to the parental diversity, the indirect choices made to optimize common nucleotides and $\Delta {\text{G}}_{\text{nn}}^{\circ }$ are unlikely to lead to runs. With less freedom, it is harder to optimize diversity. We do see that while the *f*_1 _plan is diversity-optimal for 0 and 4 mutations, it is not for 8 mutations, and the diversity-optimal plan spreads mutations out more. We also observe that the N-terminal region is so diverse that no run is produced there even with 8 mutations. Diversity optimization thus tends to create runs that are more evenly distributed in the large C-terminal region.

**Figure 9:**
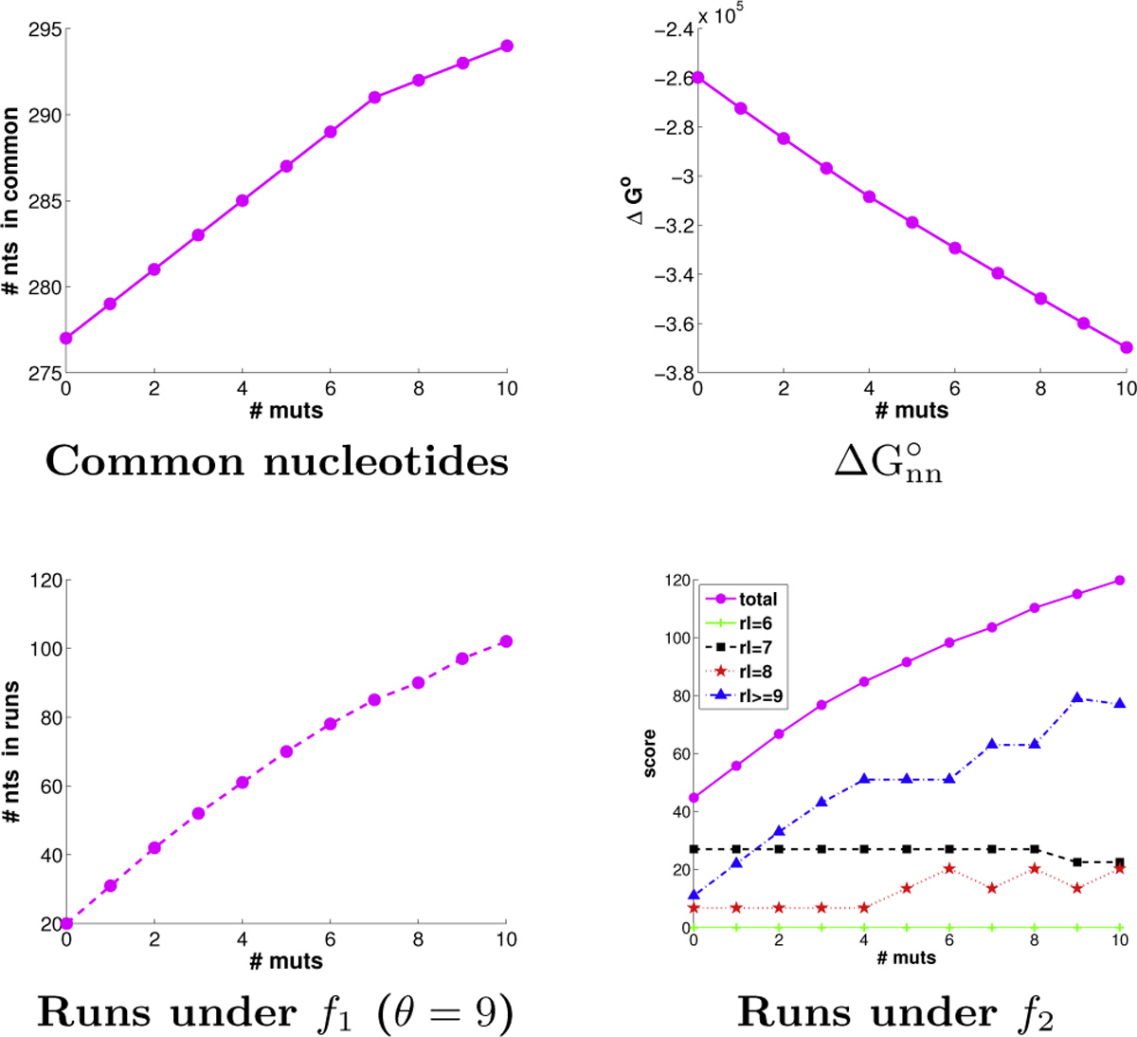
**DNA polymerase scoring trends.** The scoring trends for the DNA polymerases are shown as in Figure 5.

Figure 9 illustrates the effectiveness of allocated codons and mutations in terms of the optimizing the different objectives. We again see linear trends for the four objectives (as discussed with GAR transformylases, diversity is not directly comparable over different library sizes). Thus even with these two extremely diverse parents, it is possible to select mutations according to a specified objective, and our algorithm does so.

**Figure 10:**
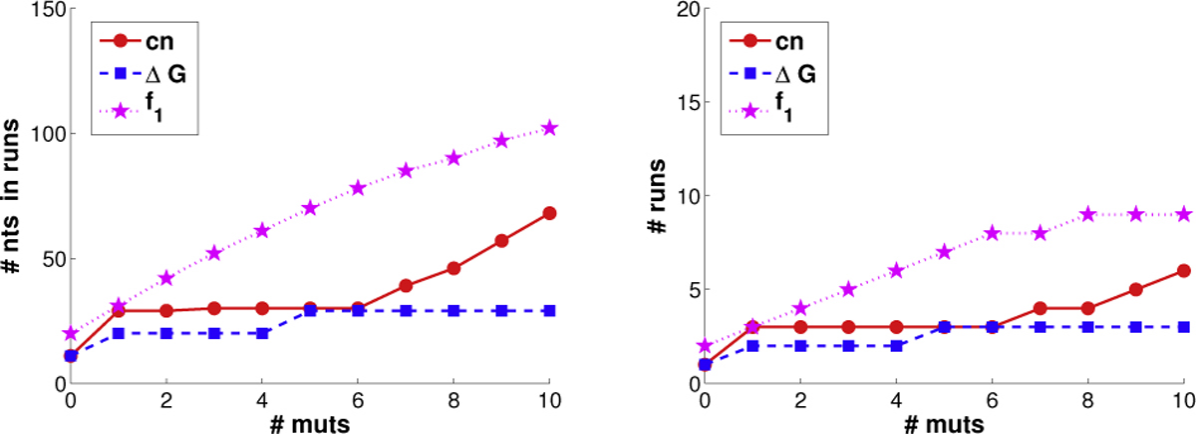
**DNA polymerase run trends.** The run trends for the DNA polymerases are shown as in Figure 6.

Figure 10 illustrates how well the different objectives do at producing runs, and how many nucleotides comprise those runs. For the sake of space (and as with GAR transformylases), we only illustrate under a 9 nt threshold for a homologous region to count as a run, but we found exactly the same trends with other thresholds from 7 to 12. Once again, explicitly optimizing for runs proves to be much more effective at producing runs (and more nucleotides in them) than does indirectly optimizing for runs by overall nucleotide identity or the nearest-neighbor approximation to change in free energy of annealing. With these diverse parents, the indirect objectives do not happen to produce many runs or nucleotides in runs; in fact, they produce almost no runs of longer lengths (e.g., 12, not shown), even with 10 mutations.

**Figure 11:**
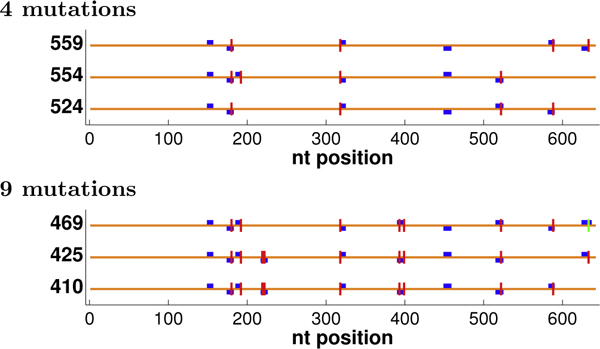
**DNA polymerase plans with different diversity values.** The plans for DNA polymerases with different diversity values (as in Figure 7).

Even with such diverse parents, there is sufficient freedom in the codon and mutation choices that different run-optimal plans yield different levels of chimera diversity. Figure 11 illustrates some such plans, at two mutation levels. The three plans at the same mutation level have the same number of runs but increasing diversity (and increasingly more even distributions of run) from top to bottom.

### Beta-lactamases

Our third case study examines the effect of wild-type sequence identity. Beta-lactamases, which hydrolyze the beta-lactam found in certain antibiotics (e.g., penicillin), have been the object of much chimeragenesis work, including DNA shuffling [1] and site-directed methods [11]. We previously developed a multiple sequence alignment (272 residues and gaps) of diverse beta-lactamases [15]. For the present study, we considered (a) the common beta-lactamase targets TEM-1 (*E. coli*) and PSE-4 (*Pseudomonas aeruginosa*) (42% amino acid identity); (b) the even more diverse pair from *P. aeruginosa *and *Bacillus licheniformis *(26% id); (c) the more similar pair from *E. coli *and *Proteus mirabilis *(47% id).

**Figure 12:**
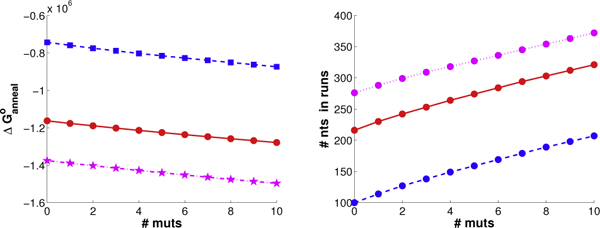
**Beta-lactamase objective function trends.** Shown are the objective function trends (left: $\Delta {\text{G}}_{\text{nn}}^{\circ }$; right: *f*_1 _at *θ *= 9) for beta-lactamase plans with 0 to 10 mutations, for pairs of beta-lactamase parents from (red) *E. coli *and *P. aeruginosa*, (blue) *P. aeruginosa *and *B. licheniformis*, and (magenta) *E. coli *and *P. mirabilis*.

Optimizing the wild-type amino acid sequences for common nucleotides yields DNA identity of (a) 70%, (b) 61%, and (c) 73%. These numbers are somewhat borderline for standard DNA shuffling. They do result in some runs, though generally fewer than when directly optimizing for runs, a trend that widens with more mutations. Optimizing for runs yields the same behavior as observed for the previous two cases; due to lack of space, we only present the free energy score and the number of nucleotides in runs under the *f*_1 _metric (Figure 12). We again see the linear tread for both objectives with increasing mutations from 0 to 10. The actual energy score and run score both depend on parental sequence identity, with the same ranking on both metrics.

## Conclusion

DNA shuffling is a staple of protein engineering, and we have demonstrated that our new algorithms can substantially improve the expected productivity of an experiment. Even without performing any mutations, we are able to allocate codons to better form runs. By performing a small number of conservative substitutions, not expected to significantly affect stability or activity, we generally are able to increase the number of runs and the number of nucleotides in runs, linearly with the number of substitutions. Finally, since we are establishing runs whose lengths are sufficient to promote regular recombination, we can enhance our optimization to account for properties of the resulting chimeric library. Future directions include extending run optimization to incorporate the type of potential underlying $\Delta {\text{G}}_{\text{nn}}^{\circ }$ (i.e., accounting for differences in nucleotide content), to optimize multiple parents simultaneously, and to integrate CODNS within our Pareto-optimization framework [29] in order to optimize productivity of shuffling in concert with other properties. While both extensions will increase the computational expense, the resulting gain in experimental efficiency could be well worth it. In summary, our methods yield a new approach to DNA shuffling that supports substantially more diverse parents, is more deterministic, and generates more predictable and more diverse chimeric libraries.

## Competing interests

The authors declare that they have no competing interests.

## Authors' contributions

LH, AMF, and CBK developed the approach; LH, and CBK designed the algorithms, LH implemented the algorithms and collected the results; LH, AMF, and CBK analyzed the results and wrote the paper. All authors read and approved the final manuscript.
